# (*R*)-(-)-Ketamine: The Promise of a Novel Treatment for Psychiatric and Neurological Disorders

**DOI:** 10.3390/ijms25126804

**Published:** 2024-06-20

**Authors:** Hana Shafique, Julie C. Demers, Julia Biesiada, Lalit K. Golani, Rok Cerne, Jodi L. Smith, Marta Szostak, Jeffrey M. Witkin

**Affiliations:** 1 Duke University School of Medicine, Durham, NC 27710, USA; 2 Indiana University-Purdue University, Indianapolis, IN 46202, USA; jucweiss@iu.edu (J.C.D.); biesiadix@gmail.com (J.B.); 3 Department of Chemistry and Chemical Biology, Northeastern University, Boston, MA 02115, USA; l.golani@northeastern.edu; 4 Laboratory of Antiepileptic Drug Discovery, Ascension St. Vincent Hospital, Indianapolis, IN 46260, USA; rok_cerne@yahoo.com (R.C.); jodi.smith@ascension-external.org (J.L.S.); 5 Department of Psychology, SWPS University, 03-815 Warsaw, Poland; martaszostak2003@icloud.com; 6 Departments of Neuroscience and Trauma Research, Ascension St. Vincent Hospital, Indianapolis, IN 46260, USA

**Keywords:** major depressive disorder, rapid-acting antidepressant, inflammation, ketamine, (*R*)-ketamine, substance use disorder

## Abstract

NMDA receptor antagonists have potential for therapeutics in neurological and psychiatric diseases, including neurodegenerative diseases, epilepsy, traumatic brain injury, substance abuse disorder (SUD), and major depressive disorder (MDD). (*S*)-ketamine was the first of a novel class of antidepressants, rapid-acting antidepressants, to be approved for medical use. The stereoisomer, (*R*)-ketamine (arketamine), is currently under development for treatment-resistant depression (TRD). The compound has demonstrated efficacy in multiple animal models. Two clinical studies disclosed efficacy in TRD and bipolar depression. A study by the drug sponsor recently failed to reach a priori clinical endpoints but post hoc analysis revealed efficacy. The clinical value of (*R*)-ketamine is supported by experimental data in humans and rodents, showing that it is less sedating, does not produce marked psychotomimetic or dissociative effects, has less abuse potential than (*S*)-ketamine, and produces efficacy in animal models of a range of neurological and psychiatric disorders. The mechanisms of action of the antidepressant effects of (*R*)-ketamine are hypothesized to be due to NMDA receptor antagonism and/or non-NMDA receptor mechanisms. We suggest that further clinical experimentation with (*R*)-ketamine will create novel and improved medicines for some of the neurological and psychiatric disorders that are underserved by current medications.

## 1. Introduction

**Figure 1:**
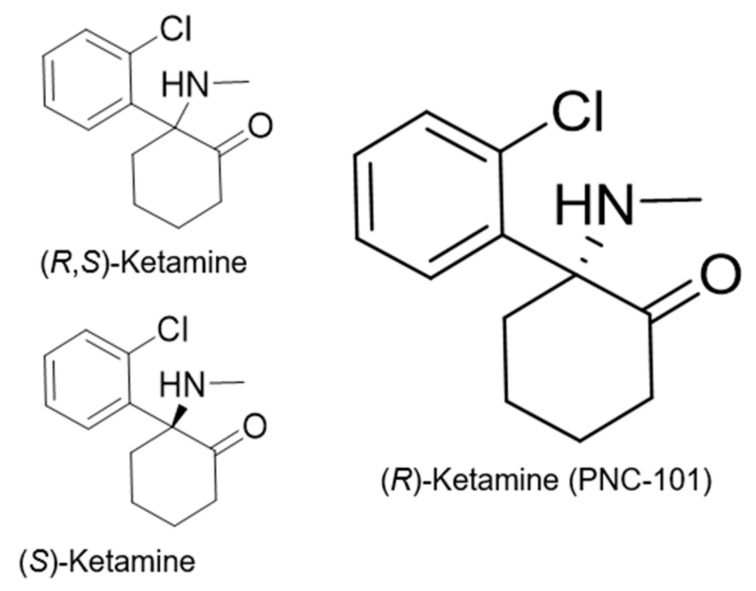
Structures of racemic ketamine and its (*R*)- and (*S*)-enantiomers.

Ketamine (Figure 1) was synthesized in 1962 with the goal of creating a compound with less psychotomimetic effects than its congener phencyclidine (PCP). The pharmacology of ketamine and its (*S*)-(+)- and (*R*)-(-)-isomers and metabolites (Figure 1) have been intensively reviewed [1]. Ketamine has major pharmacological effects as an anesthetic, as it is widely used. Its use as an analgesic also continues under some circumstances. Ketamine also has antidepressant and anti-inflammatory properties [1]. The side effects of ketamine include psychotomimetic effects like that of PCP, cognitive and motoric impairment, and damage to brain cells with repeated dosing [1,2]. Ketamine is also a drug of abuse and is associated with drug overdose [1,3].

The present paper provides a review of key aspects of the pharmacology of (*R*)-ketamine that set the stage for updates in the experimental literature that have not been given comprehensive review as of yet. The literature shows its unique pharmacological properties that we believe could make it an important new medicine in psychiatry and neurology. The primary rationale for this position is based upon the data that highlight the efficacy of (*R*)-ketamine in animal models that are used to predict treatments for major depressive disorder (MDD), for the subset of MDD termed treatment-resistant depression (TRD), for substance use disorder (SUD), and other disease states (Section 5). Our focus on (*R*)-ketamine derives from the data in preclinical and clinical studies that document that its side effects are, in many cases, both qualitatively and quantitatively lower than that of racemic ketamine or (*S*)-ketamine. Particular attention will be given to the comparative pharmacology of (*R*)- and (*S*)-ketamine. After discussing the data surrounding the efficacy and side effects of (*R*)-ketamine, we will discuss other compounds that are being considered as rapid-acting antidepressants (Section 3).

Although the seminal data on these compounds will be discussed, a primary goal of the present review is to provide an updated literature analysis. As of 21 March 2024, there were 1862 papers published on (*S*) vs. 282 on (*R*)-ketamine [4] with the first paper on the isomers published by White and colleagues [5]. Large increases in the number of publications from that time have occurred, with 2015 being an inflection point for (S)-ketamine, and its approval as a medicine was granted in 2019. The increase in the rate of publications for (*R*)-ketamine began in 2016 after the first antidepressant differentiations of the isomers of ketamine [6,7]. The peak year for the number of research reports was 2023 for (*S*)-ketamine, with 436 papers, and 2022 for (*R*)-ketamine, with a total of 49 papers in that year. Since the present paper is an update on the literature on (*R*)-ketamine, the reader is referred to earlier overviews for historical perspective [1,2,8,9].

The overview and discussion of the data on (*R*)-ketamine follows an outline that emphasizes the primary clinical foci that have been placed upon this compound. (*R*)-ketamine is currently in clinical development only for MDD, in particular TRD. We review these data first. After that, we provide a brief update of the major areas of drug discovery and development that deal with other potential rapid-acting antidepressants to place the data on (*R*)-ketamine into perspective. Additional preclinical work on (*R*)-ketamine has been on substance use disorder and this area, still in the preclinical stage, is reviewed next. The possibility for additional therapeutic uses of (*R*)-ketamine is discussed next, including the psychiatric and neurology domains. Given the data reviewed on potential efficacy, this manuscript next turns to the potential side effects and safety issues that might be presented with the therapeutic use of this compound. Finally, the present review provides a very rough overview of the experimental literature that has and continues to help define its mechanisms of action. This later section is not meant to be exhaustive.

Note: the rationale for the present overview of the pharmacology of (*R*)-ketamine is to provide, first, a basic review of the state of the science that has been previously disclosed in the literature in primary papers and in reviews. The main impetus for the current review paper, however, is to update the reader regarding new areas of experimental inquiry that have yet to be reviewed for this compound. Therefore, for the primary overview of pharmacology, we point the reader to the prior literature but also to excellent high-level research reviews. For the later goal of summarizing and discussing new experimental data, primary data sources are provided.

## 2. Major Depressive Disorder (MDD) and Treatment-Resistant Depression (TRD)

The Diagnostic and Statistical Manual of Mental Health (DSM-5-TR) definition for Major Depressive Disorder (MDD) is a period of two weeks or more of depression or lack of interest/pleasure found in most daily activities and represented by symptoms such as difficulty sleeping/insomnia, changes in weight or appetite, agitation, lack of concentration, a poor sense self-worth, or persistent thoughts of death/suicide [9,10]. MDD affects millions of people worldwide and often inflicts devasting consequences on the lives of the patient, family members, and society [10]. Prevalence estimates in the United States alone show over 20 million adults and 5 million adolescents in 2021 that were affected. Prevalence rates were higher among adults aged 18–25 years old. Nearly 75% of adults and just over 44% of adolescents sought some form of treatment for their condition [10].

Although medications have existed for many years (antidepressants) [9,11], there are multiple reasons that led to the 2019 U.S. FDA approval of (*S*)-ketamine for the treatment of patients when given in conjunction with standard-of-care (SOC) antidepressants like monoamine uptake inhibitors. One pressing reason for the approval of (*S*)-ketamine is that about 30% of MDD patients do not respond to SOC antidepressants—treatment-resistant depression (TRD). In addition to TRD, SOC antidepressants take weeks of daily administration before full antidepressant effects are produced [9].

Although case observations in 1964 showed that ketamine could be be antidepressant [12], it was not until 1990 that Trullas and Skolnick deduced that the blockade of NMDA receptors (NMDARs), a principal mechanism of action of ketamine [1], might be an alternative antidepressant mechanism to the prevailing and long-standing monoamine hypothesis [13]. They hypothesized that (1) certain stressors (inescapable) disrupt long-term potentiation (LTP), and (2) since LTP is dependent on NMDAR activation, and (3) since inescapable stressors produce a behavioral phenotype that can be reversed by clinically effective antidepressants, NMDAR antagonists might produce antidepressant-like effects in these behavioral stress models. Trullas and Skolnick [13] affirmed this hypothesis with experimental data using a number of NMDAR antagonists acting at different sites on the receptor complex. Thus, the NMDAR hypothesis of antidepressant drug action was born [14]. A clinical verification of the NMDAR hypothesis was published in 2000, where it was demonstrated that an intravenous (i.v.) infusion of a non-anesthetic dose of ketamine could induce antidepressant effects in patients [15].

A critical systematic replication of the Berman et al. study in TRD patients showed that about 50% of patients responded to a 40 min i.v. ketamine infusion and that the antidepressant effects lasted multiple days in some patients [16]. Since then, multiple systematic replications of the antidepressant effects of ketamine have been reported [1,2,9]. Differentiating itself from the monoamine-based antidepressants, ketamine infusion rapidly acted as an antidepressant, and, importantly, was effective in TRD patients [16]. Further studies with ketamine have documented its efficacy against some MDD symptoms that do not readily resolve with SOC drugs and that ketamine also has antidepressant effects in bipolar disorder [17]. Ketamine and now other compounds (Section 3) are considered a new class of antidepressants called rapid-acting antidepressants [18]. The efficacy of ketamine infusion to resolve TRD in some patients was life-changing and lifesaving and led to the off-label use of ketamine for TRD patients in infusion clinics to this day. A major issue still unresolved is the optimization of ketamine dosing so as to not generate relapse into depression [19,20,21].

(*S*)-ketamine was developed as an antidepressant based upon the data from the evolving clinical studies on racemic ketamine (typically by i.v. dosing). (*S*)-ketamine was approved for use in the United States in 2019 for TRD as an adjunct therapy (in nasal spray formulation as Spravato^@^) to ongoing SOC monoamine-based antidepressants [22]. Since that time, (*S*)-ketamine has demonstrated efficacy and tolerability when given as approved with in-patient visits. Recent estimates have also indicated a marked uptake in the use of this medication, with a 72% increase in sales from Q1 2023 to Q1 2024. Spravato is currently approved in over eight countries [21].

Animal models used to predict antidepressant effects in humans have been misaligned by a number of writers and by pressure from animal rights groups and public policy makers [23]. However, (*R,S*)-ketamine and (*S*)-ketamine are proven examples of how new medicines for depression can arise through the thoughtful use of data from these model systems [13]. Following from these drugs are multiple other new drugs and potential new medicines like (*R*)-ketamine (see [9,14,24,25] and Section 3 of the present paper). Models like the forced-swim test, if calibrated properly, are simple, rapid, and predictive methods for helping researchers identify novel and improved antidepressant medicines that can also help guide human dosing [26]. Further, rodents know how to swim and under laboratory use, the forced swim assay did not result in increases in standard plasma stress markers (corticosterone; JMW, unpublished data).

Laboratory studies, using rodent models, from Kenji Hashimoto’s laboratory provided the first data that suggested that the (*R*)-isomer of ketamine might be an improved choice for MDD and TRD therapy. Using chronic models of stress to induce depression-like signs in rodents, they showed that (*R*)-ketamine was more potent and longer lasting than (*S*)-ketamine in attenuating these behavioral phenotypes [7,27,28]. What was striking about these observations is that (*R*)-ketamine is somewhat less potent than (*S*)-ketamine as an NMDAR antagonist [1]. Equally important were the preclinical observations that (*R*)-ketamine presented with a lower side-effect burden in these experiments than that of (*S*)-ketamine [6,27,29]. Taken together, the efficacy (as noted above) and side-effect data (Section 6) on (*R*)-ketamine suggested that (*R*)-ketamine might be a more ideal antidepressant than (*S*)-ketamine [30].

Based upon these preclinical findings, (*R*)-ketamine was initiated into clinical development (Phase 2) for TRD by Perception Neurosciences [30]. Two open-label clinical studies demonstrated efficacy in TRD patients [31] and in depression in bipolar disorder [32]. In both studies, there was minimal or no dissociative or psychomimetic effects reported for (*R*)-ketamine. The clinical data are consistent with prior human studies that show (*R*)-ketamine to produce different subjective effects than those of (*S*)-ketamine [33,34]. Preclinical findings have also pointed out notable qualitative differences in the behavioral effects of the ketamine stereoisomers, including predictors of subjective effects (Section 6).

Perception Neurosciences has conducted multiple clinical studies with (*R*)-ketamine (PNC-101) [35]. Efficacy signals in TRD patients were reported by the company in Phase 2 studies with two i.v. doses but the compound failed to reach the company’s a priori primary endpoints [36]. Further scrutiny of these data showed significant improvement in depression scores up to two weeks after a single i.v. dose. Forty three percent of patients achieved remission at 24 h [35]. Additional work is ongoing by the company in the development of PNC-101 in Japan for TRD and in studying subcutaneous formulations [35].

Prior reviews of the antidepressant effects of ketamine and its isomers are available [1,2,9,25,30,37,38,39,40].

A number of studies have been conducted in the past few years that have confirmed the antidepressant-like effects of (*R*)-ketamine and extended the generality of prior observations. The data from these studies have elaborated on the conditions under which (*R*)-ketamine produces antidepressant-like efficacy, provided additional comparative data with (*S*)-ketamine, and expanded the knowledge base on putative mechanisms of action. The latest of these studies are highlighted here.

Using C57BL/6J mice, it was shown that (*R*)-ketamine produced anti-anhedonic and anti-apathy effects for up to 7 days, (*S*)-ketamine was shorter lived in its effects (3 days) [41]. These findings verified prior data from Hashimoto’s lab on the long-lived effects of (*R*)-ketamine and, importantly, extended the scope of action of (*R*)-ketamine to a model of chronic unpredictable mild stress. Another new study on the effects of (*R*)-ketamine investigated prophylactic treatment for two procedures that induce depressive-like behaviors in mice. In this study, (*R*)-ketamine attenuated depressive-like behaviors induced by lipopolysaccharide and by chronic restraint stress when given either 6 or 7 days prior to application of the stressors [42].

(*R*,*S*)-ketamine and (*S*)-ketamine previously demonstrated an ability to synergize with the antidepressant-like behavioral effects of other antidepressants, including SOC drugs like fluoxetine [43]. In a recent study, the antidepressant-like effects of M-5MPEP, a negative allosteric modulator of mGlu5Rs, were enhanced by the addition of (*R*)-ketamine. The study evaluated the effects 24 h after four doses using the splash test (a behavioral marker of apathy), the sucrose preference test (anhedonia), and the tail-suspension test, which is a general predictive model of antidepressant efficacy [44,45].

Two studies on timing behavior have recently been reported that present data with implications for the antidepressant effects of (*R*)-ketamine. Popik and colleagues hypothesized that time estimation studies would provide insight into the antidepressant actions of drugs since MDD patients report a slowed time perception. In one study, (*S*)-ketamine shifted time estimation curves indicative of time underestimation; (*S*)-ketamine also decreased the temporal discrimination accuracy of temporal discrimination. In contrast, (*R*)-ketamine did not affect the timing behaviors of rats, as (*R*)-ketamine produced pro-cognitive effects—decreases in incorrect responses and increases in accuracy. The authors concluded that while the time underestimation produced by (*S*)-ketamine might be a marker of its antidepressant efficacy, (*S*)-ketamine, in contrast to (*R*)-ketamine, produced marked disruptions in behavior [46]. A study by Malikowska-Racia et al. [47] showed that the antidepressant fluoxetine, and (*R*)- and (*S*)-ketamine modified the timing behavior of rats under a differential reinforcement of low-rate (DRL) schedule typifying antidepressant drugs [48]. In that study, (*R*)-ketamine differed from (*S*)-ketamine and fluoxetine in time perception estimates. Interestingly, the authors also reported the unanticipated observation that the effects of fluoxetine on the performance of the rats under the DRL schedule was potentiated by naloxone, a potentially clinically meaningful finding.

In contrast to the most recent data summarized above on (*R*)-ketamine, another set of experiments failed to demonstrate an antidepressant-like behavioral signature for (*R*)-ketamine. Rats undergoing chronic stress demonstrated antidepressant-like anti-immobility effects when given (*S*)-ketamine; this effect was not observed with (*R*)-ketamine [49]. EEG studies in these rats also showed antidepressant-associated patterns with (*S*)- but not with (*R*)-ketamine. Follow-up studies in rats continued to document differential effects of (*R*)- and (*S*)-ketamine on EEG measures [50]. Whether these negative preclinical findings on antidepressant-like efficacy are due to species (mice have been the dominating research subjects in studies of (*R*)-ketamine), the use of only one dose of the compounds, and/or other procedural differences across studies, will have to be determined.

## 3. Other Potential Rapid-Acting Antidepressants

Primarily driven by the data on racemic ketamine and (*S*)-ketamine as antidepressants, a multitude of additional compounds have been interrogated in the laboratory for their potential as rapid-acting antidepressants. Table 1 summarizes the primary compounds that have undergone experimental scrutiny, including approved medications. Given that (*R*)-ketamine is currently under development as a rapid-acting antidepressant, this brief summary of the competitive landscape is meant to place (*R*)-ketamine in perspective as a developmental candidate for depression. A discussion of these data is available [2,9,24,25,30,51,52].

Although most compounds in this table are NMDA receptor antagonists, two compounds are potentiators of GABAARs (brexanolone and zuranolone), both for post-partum depression. Two additional classes of drugs are being considered as well—psychedelics and non-NMDA glutamate modulators (AMPA and mGlu2/3).

## 4. Substance-Use Disorder (SUD)

**Figure 2:**
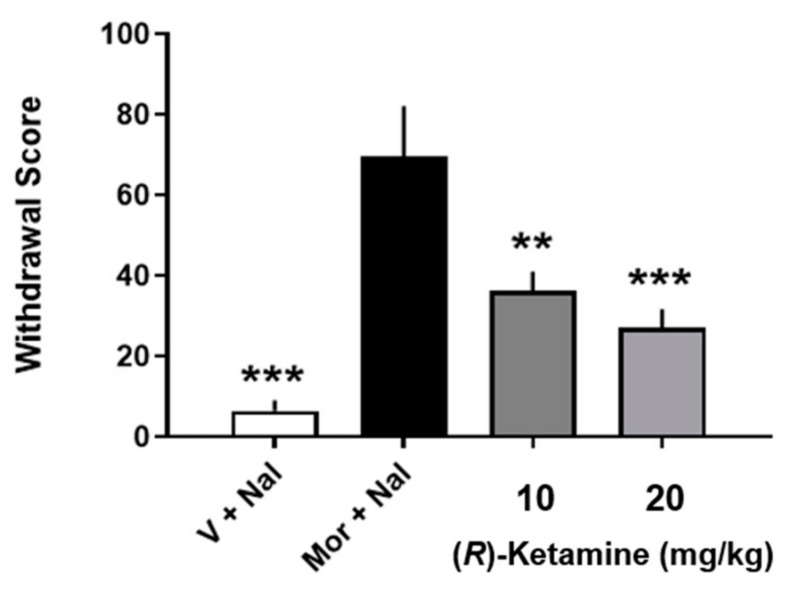
Effects of (*R*)-ketamine on overall withdrawal scores in rats undergoing naloxone-precipitated withdrawal from subchronic morphine. Each bar represents the mean + SEM of data from eight rats V: vehicle; Nal: naloxone; R-ket: R-ketamine; ** *p* < 0.01, *** *p* < 0.001 compared to mor + nal group by Newman–Keuls multiple comparison test. Figure is modified from [54] with permission.

**Figure 3:**
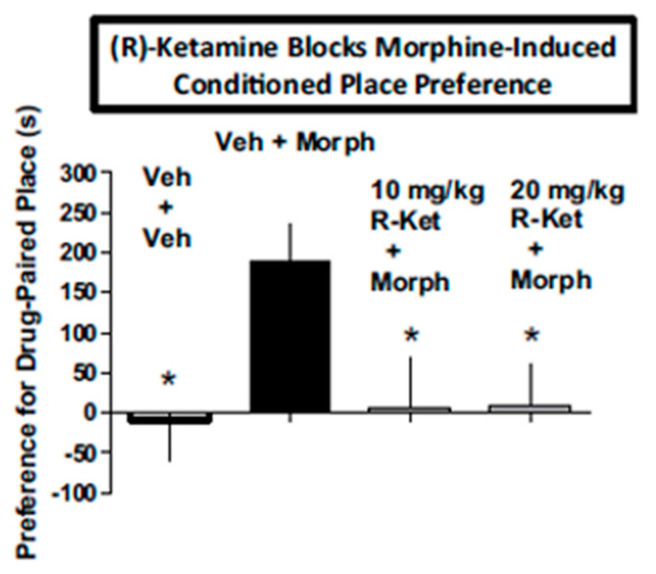
(*R*)-ketamine (10 and 20 mg/kg) blocks the conditioned place preference engendered by 5 mg/kg morphine. * *p* < 0.05 compared to vehicle + morphine treatment by Dunnett’s test. Each bar represents the mean + SEM of data from 9 to 13 mice/group. Figure is modified from [54] with permission.

SUD is a highly prevalent disorder that has a huge impact upon individual lives and society and involves the use of prescription and non-prescription drugs for uses and quantities outside the boundaries of regulatory guidance. SUD comprises the areas of drug use concerning tolerance, physical dependence, drug abuse, and diversion [53]. Prior studies have documented the potential value of (*R*)-ketamine as an aid in SUD [54]. (*R*)-ketamine attenuated withdrawal signs in morphine-dependent rats (Figure 2) and blocked the subjective effects of morphine in conditioned place preference studies (Figure 3). Subsequent studies showed that (*R*)-ketamine prevented tolerance to effects of ethanol on some behavioral measures [55]. Taken together with the non-intrusive side-effect profile of (*R*)-ketamine observed in these studies and elsewhere, which includes its predicted low abuse liability (Section 6), (*R*)-ketamine is suggested to be a potentially novel and safe treatment alternative for some aspects of SUD.

## 5. Other Potential Therapeutic Applications

Recent experimental data have suggested additional therapeutic applications for (*R*)-ketamine. Table 2 shows a list of these ideas along with the rationale from the experimental findings that are discussed in this section. Prior reviews of this area can be found [56,57].

Racemic ketamine has long been known for its anesthetic properties [1] and is currently utilized in the management of pain in some cases where other therapies are not effective [58]. The potential contribution of (*R*)-ketamine to the efficacy of racemic ketamine was assessed in healthy volunteers by pharmacokinetic-pharmacodynamic analyses. While (*S*)-ketamine contributed to both alterations in perception of drug effects (dissociative effects) and pain perception, (*R*)-ketamine did not alter perception or pain detection [59]. The authors concluded that the antinociceptive effects of ketamine are associated with its dissociative effects. While these data support the existing literature on the distinctions between (*R*)- and (*S*)-ketamine in the production of dissociative reactions, additional studies would help clarify whether there is a role for these isomers as an antinociceptive agent.

Inflammation is highly associated with and causal in pain generation and maintenance [60]. (*R*)-ketamine has been shown to reduce inflammatory responses. Organ damage and lethality could be attenuated by (*R*)-ketamine when given prior to and during lethal inflammatory induction induced by cecal ligation and puncture in mice. This regimen of (*R*)-ketamine resulted in reductions in multiple inflammatory markers, organ failure markers, and morphological damage from sepsis [61].

An example of how this anti-inflammatory property could have other therapeutic uses comes from data from this research team. Bacterial lipopolysaccharide (LPS) was given to produce endotoxemia in mice, with increases observed in a host of inflammatory biomarkers that were also detected in the prefrontal cortex (PFC). (*R*)-ketamine given prior to and just after LPS reduced these changes and the inflammation-induced deterioration in behavior and cognitive function [62]. As this LPS mouse model has some validation as a predictor of the efficacy of anti-delirium medications, the authors considered (*R*)-ketamine as a potential new drug in that therapeutic domain. Wang and colleagues have also provided findings documenting the value of (*R*)-ketamine in experimental autoimmune encephalomyelitis in mice, suggesting its possible prophylactic use in multiple sclerosis (MS) [63].

A number of other data sets agree with the potential value of (*R*)-ketamine in cognitive disorders, as noted above in the work of Zhang et al. [62]. Examples of these cognitive-protecting effects can be found [64]. Other experiments were able to differentiate the cognitive activity of (*R*)-ketamine from the inactivity of (*S*)-ketamine [65], although under other conditions, such as pain-induced cognitive impairment, (*S*)-ketamine can be protective as well [66].

That (*R*)-ketamine might have beneficial effects in neurodevelopmental disorders has also been experimentally considered. Tan and colleagues have reported a protective effect of prophylactic (*R*)-ketamine in rodent models generating schizophrenia-like behaviors in offspring after maternal immune activation [67]. Concordant findings were subsequently reported [68].

## 6. Side Effects

(*R*,*S*)-ketamine produces a variety of side effects along with tolerability and safety issues including dissociative reactions, psychotomimetic effects, impairment of memory, and drug abuse [1]. The most commonly reported side effects of (*S*)-ketamine, or esketamine, include dissociation, nausea, vertigo, headache, and dizziness [69,70]. In 2022, the Food and Drug Administration (FDA) informed physicians about the potential tolerability and safety issues associated with esketamine nasal spray [71]. Due to the risks associated with Spravato^®^ such as sedation, dissociation, and the risk of abuse or misuse, stringent safety measures are in place. These include Boxed Warnings on its label and adherence to a Risk Evaluation and Mitigation Strategy. The dispensing and administration of esketamine must occur exclusively in certified healthcare centers. As such, it is crucial to consider the use of the (*R*)-(-)-ketamine isomer as a possible alternative therapeutic agent where outpatient use might be possible.

One of the striking pharmacological characteristics of (*R*)-ketamine that differentiate it from its racemate, and (S)-isomer are its novel, benign side effects. Table 3 summarizes the predominant differentiators that are discussed in this section. Accumulating clinical [33,34] and preclinical data [6,27,29] have suggested that (*R*)-ketamine might provide a better overall side-effect pharmacological profile than that of (*S*)-ketamine.

A comparative study of the behavioral side effects of (*S*)- and (*R*)-ketamine in rats showed marked quantitative and qualitative differences when given at the same dose levels. (*S*)-ketamine induced increases in locomotion and muscle rigidity, whereas (*R*)-ketamine did not. Although both isomers produced some stereotypies, dystonia, and uncoordinated movements, the (*S*)-isomer produced more profound alterations and was more potent [54]. Motor side effects of (*R*)-ketamine were also milder or absent in rats given (*R*)- vs. (S)-ketamine, and unlike (*S*)-ketamine, (*R*)-ketamine did not disrupt ongoing behaviors of rats [72].

EEG recordings in five healthy volunteers showed that (*R*)-ketamine produces less overall slowing and an absence of the large slow wave complexes compared to the (*S*)-isomer and the racemic mixture [5]. Indeed, it was reported that both racemic ketamine and (*S*)-ketamine produced significant psychopathology and neurocognitive impairment compared to placebo without significant differences between these two ketamine forms [73]. Given the reports of milder side effects generated by the (*R*)-isomer, the authors indicated that additional human data on the (*R*)-isomer would be welcome.

The high liability for abuse of racemic ketamine in humans and experimental animals is well known [1,3]. Data have demonstrated that while (*S*)-ketamine is readily self-administered by rats, (*R*)-ketamine was not when comparing antidepressant-producing doses of the two isomers [74]. Other behavioral effects associated with drug abuse liability (locomotor enhancement, sensitization, conditioned place preference, and augmentation of dopaminergic activity in the medial prefrontal cortex (mPFC)) were also produced by (*S*)- but not by (*R*)-ketamine. Receptor binding analyses showed qualitative differences in activity in the mPFC of rats that might be associated with the divergent side effects of (*S*)-ketamine [74].

**Figure 4:**
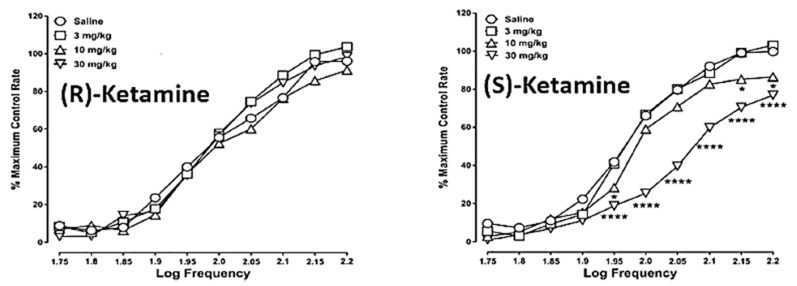
Frequency-rate response curves for the effects of (*R*)-ketamine (**left panel**) or (*S*)-ketamine (**right panel**) in rats responding under a schedule of electrical stimulation of the medial forebrain bundle (intracranial self-stimulation). Values represent the mean normalized response rate (% of maximum control responding) across 10 frequency presentations (1.75–2.20 log/Hz) of 10 rats. Error bars are omitted for clarity. Significant differences compared to vehicle at the respective frequencies are denoted by asterisks (* *p* < 0.05; **** *p* < 0.0001; *N* = 10). Data are from [54] with permission.

Data in patients with TRD [31,35] and bipolar depression [32] showed low or no levels of dissociative and psychotomimetic effects when given (*R*)-ketamine. In rats, (*S*)- but not (*R*)-ketamine shifted the frequency/effect function for intracranial self-stimulation (ICSS) down and to the right, indicative of a dysphoric-like activity (Figure 4).

**Figure 5:**
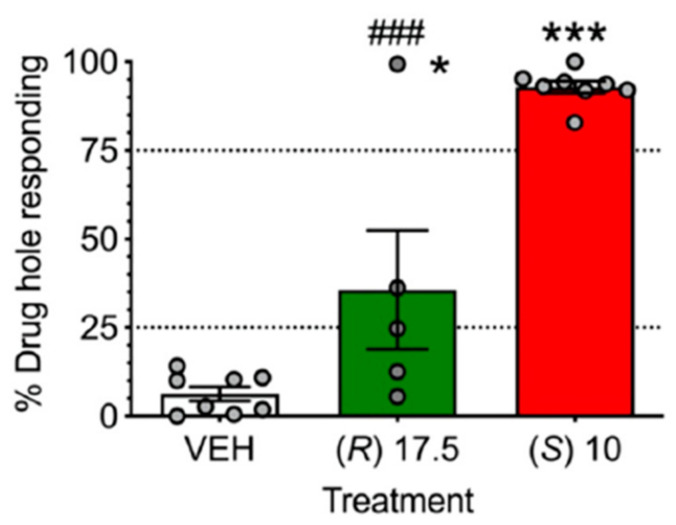
(*R*)-ketamine did not fully substitute for the training dose of (*S*)-ketamine in (*S*)-ketamine-trained rats. Ns for vehicle and (*S*)-ketamine were eight, whereas eight rats were tested with (*R*)-ketamine (17.5 mg/kg). Symbols: * *p* < 0.05, *** *p* < 0.001 vs. vehicle, ### *p* < 0.001 vs. training dose of (*S*)-ketamine. Dotted lines represent 25 and 75% accuracy. Data are from [76] with permission.

**Figure 6:**
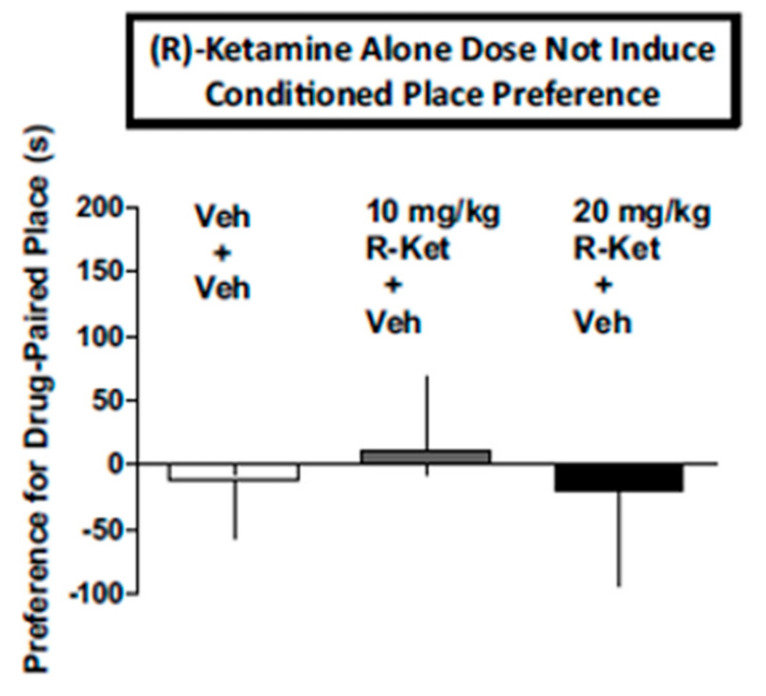
(*R*)-ketamine at 10 and 20 mg/kg does not induce a conditioned place preference when given alone (+vehicle). Each bar represents the mean + SEM of data from 10 mice/group. Veh: vehicle or saline; R-ket: (*R*)-ketamine. Data are from [54] with permission.

The first report using a model predictive of human subject effects, drug discrimination [75], recently showed qualitative and quantitative differences in the discriminative stimulus effects of (*S*)- and (R)-ketamine in rats trained to discriminate different isomers of ketamine. In rats trained to discriminate (*S*)-ketamine, (*R*)-ketamine did not fully substitute (Figure 5). These subjective effect differences appear to correspond to the abuse liability differences of (*R*)- vs. (*S*)-ketamine discussed above. In contrast to the abuse-associated behavioral and neurochemical actions of (*S*)-ketamine as discussed above, (*R*)-ketamine at doses that significantly attenuated withdrawal signs in morphine-dependent rats (Figure 2) and morphine-induced place preference (a marker of drug abuse) (Figure 3), did not produce conditioned place preference when given alone (Figure 6). These data point again to the lower predicted abuse liability of (*R*)- compared to (*S*)-ketamine.

## 7. Mechanisms of Action

Reviews of the literature on the mechanisms of action of the antidepressant effects of ketamine and that of its isomers are available [1,2,25,30,37,38,77,78,79,80,81,82,83,84]. New examinations on the mechanistic and therapeutic roles for NMDAR antagonism have also been published [85]. There are currently two major schools of thought on the mechanisms associated with the antidepressant-like effects of ketamine and its isomers: NMDAR antagonism and non-NMDAR antagonist mechanisms. In our view, these ideas are not necessarily mutually exclusive. The well-described pathway for inducing the antidepressant and antidepressant-like effects of ketamine and its isomers is the transduction of antidepressant-associated biochemical alterations that are triggered by the blockade by ketamine of the NMDAR ion channel [2,9,18,86,87]. The resultant enhancement of excitatory neurotransmission and the induction of neurotrophic factors associated with antidepressant effects have been a long-standing mechanistic model. The ideas of non-NMDA mechanisms of action derive from a number of sources that include the potential for metabolites (non-NMDAR antagonists) to produce the antidepressant-like effects of ketamine [24,88,89] and data associating a number of non-NMDAR mechanisms of ketamine with its antidepressant efficacy [90] (Section 7).

Other data have suggested additional rationale for attributing the effects of (*R*)-ketamine to non-NMDAR mechanisms. Among the rationale for this line of thinking are (1) differences in NMDAR affinity differences of (*R*)- and (*S*)-ketamine and their comparative antidepressant-like effects [38], and (2) differences that have been observed between (*R*)- and (*S*)-ketamine [50,80,81,83,91]. Some of the newer data in this area are discussed in this section. Discussion here is not meant to be comprehensive and primarily covers newer findings comparing the two ketamine isomers. The reader is referred to the literature cited for more detail.

Mechanistic investigations into the pharmacological actions of (*R*)-ketamine have principally been conducted for its antidepressant-like biological actions. Thus, although (*R*)-ketamine might have therapeutic utility in other diseases (Section 5), the data in these ancillary areas is not as developed as for its antidepressant-related pharmacology [56,78].

Rafało-Ulińska and Pałucha-Poniewiera provided data showing differences in biochemical transduction pathways subserving the pharmacological effects of (*S*)- and (*R*)-ketamine [41]. Using chronic unpredictable stressors to induce behavioral changes in mice, they showed that the activation of TrkB receptors was involved in the antidepressant-associated effects of (*R*)- but not (*S*)-ketamine. (*S*)-ketamine, in contrast, was suggested to possibly operate through mTOR and ERK pathways and upregulate GLuA1 expression in the mPFC, as others have disclosed before (see [91] for more details on biochemical pathways for (*R*)-ketamine). The mTOR pathways were also activated by (*R*)-ketamine, but ERK phosphorylation in mPFC was not significantly changed. This research group also recently documented the ability of a partial mGlu_5_ receptor negative allosteric modulator, M-5MPEP, to augment the antidepressant-like effects of (*R*)-ketamine in mice. The authors suggested the potential of augmentation therapy with this drug combination for rapid antidepressant response in MDD or TRD patients that likely is linked to their common actions on inducing BDNF production [44]. Based upon data from electroencephalogram (EEG) recordings in rats, differences in theta power were observed between (*R*)- and (*S*)-ketamine [50]. These findings suggested that (*R*)-ketamine might have effects on hippocampal function that do not occur with (*S*)-ketamine. The authors concluded that the hippocampal activity induced by (*R*)-ketamine could be responsible for its pharmacologically beneficial effects on neural plasticity and cognitive function derived from preclinical studies (see Section 5).

Additional new data on the mechanisms by which (*R*)-ketamine transduces its antidepressant-like effects have also been disclosed. These data provide new information on the impact of (*R*)-ketamine on biological pathways not previously discussed, such as macrophage migration inhibitory factor [92], pathways associated with the microRNA mi(R)-132-5p [93,94], novel ERK-NRBP1-CREB-BDNF pathways in microglia [95], the gut–brain axis [96], and neural myelination, where (*R*)-ketamine has greater efficacy than (*S*)-ketamine [97], as well as immune function and inflammatory processes [78,98,99,100]. It is important to recognize that multiple other potential mechanisms of action might be relevant but have not been interrogated yet to as great an extent. For example, what is the differential pharmacological impact of (*R*)- vs. (*S*)-ketamine on reward-related neurotransmitters in mood-regulating brain areas or their potential differential effects on cortical activation/repression in mood circuits?

Whether the pharmacological effects of ketamine require the awareness of drug effect (e.g., dissociation) has been a source of discussion for several years now. New data on this topic, including that on (*R*)-ketamine, have recently been reported [101,102,103,104].

## 8. Conclusions

Since the initial reports from Kenji Hashimoto’s laboratory that the (*R*)-(-) isomer of ketamine was more potent and longer lasting in its ability to attenuate depression-associated phenotypes in rodent models of chronic stress than that of (*S*)-(+)-ketamine (Section 2), (*R*)-ketamine has been brought into clinical development under the name PNC-101. Exploration of the diverse pharmacological effects produced by (*R*)-ketamine has grown dramatically.

The present overview emphasizes several key features of (*R*)-ketamine that might make it a novel, valuable, new therapeutic agent in neurology and psychiatry. Clinical trial findings with PNC-101 have shown signals of antidepressant effects in TRD and bipolar disorder and new research findings continue to emphasize unique features of the pharmacology of (*R*)-ketamine that encourage further clinical study in depression (Section 2) and other areas (Section 5). Meanwhile, a plethora of other potential rapid-acting antidepressants are in various stages of development (Section 3). Data from preclinical studies have introduced a number of new potential areas of therapeutic benefit for (*R*)-ketamine (Section 4 and Section 5). New data on the tolerability and safety pharmacology of (*R*)-ketamine continue to emphasize its mild side-effect profile (Section 6). In addition, for the past several years, new data on the mechanisms of action of (*R*)-ketamine have been published (Section 7). Throughout this overview of the literature, the data have highlighted commonalities in the effects of (*R*)- and (*S*)-ketamine but have also emphasized many divergent pharmacological features.

There are several issues that should additionally be considered when reviewing the current literature on (*R*)-ketamine. Among these, we cite two here. First, it is critical to note that exacting standards of comparative pharmacology have not always utilized important convergent experimental methods [105]. For example, one aspect among others that is generally missing in the data analysis are studies comparing stress-susceptible compared to stress-resistant phenotypes. Another issue that should be taken into account involves the general bias in the literature for the use of male research subjects without comparative pharmacological evaluation in females. In the case of (*R*)-ketamine, this is likely important given the reported sex differences in studies of the glutamate system [106,107,108,109]. Indeed, the U.S. NIH has, since 2016, had a policy on the inclusion of sex as a biological variable in research studies.

Another issue to keep in mind when considering the data summarized in this manuscript is the fact that there are limited data on (*R*)-ketamine in humans and even less in target patient populations. Additional knowledge on the potential efficacy, side effects, and comparative pharmacology of (*R*)-ketamine will be gleaned as additional clinical findings are reported.

Taken as a whole, the data on (*R*)-ketamine highlight its potential therapeutic value in a number of neurological and psychiatric disorders with an improved edge over SOC agents in efficacy, tolerability, and safety pharmacology.

## Figures and Tables

**Table 1:** Rapid-acting antidepressants and newer compounds under development.

Compound	Mechanism	Disease	Predicted Safety
Brexanolone (Zulresso^®^)	GABAAR agonist	Approved as i.v. infusion for severe post-partum depression	In-hospital monitoring for sedation and other
(*S*)-Ketamine (Spravato^®^)	NMDAR antagonist and possible other	Nasal spray approved as an adjunct therapy for TRD	Generally well tolerated with in-patient dosing
Racemic ketamine intranasal (SLS-002)	NMDAR antagonist and possible other	ASIB, MDD, PTSD	Generally well tolerated with in-patient dosing
Racemic ketamine (oral formulation) KET01	NMDAR antagonist and possible other	TRD	No dissociative or psychotomimetic effects
Dextromethorphan/Bupropion (Auvelity^®^)	Low-affinity NMDAR antagonist, monoamine uptake blocker	MDD	Generally well tolerated
Zuranolone (Zurzuvae^®^) ^1^	GABAAR potentiator, Oral drug	Approved as oral medication for post-partum depression	Drowsiness, dizziness
(*R*)-Ketamine (PNC-101) ^2^	NMDAR antagonism and possible other	TRD	No dissociative or psychotomimetic effects
(*S*)-Methadone ^2^	NMDAR antagonism and possible other	MDD and TRD	Low dissociative and psychotomimetic liability
MIJ821 (CAD-927) (Onfasprodil)	Low-affinity NMDAR antagonist	TRD	Transient mild dissociative effects
Lanicemine (AZD6765)	Low-affinity NMDAR antagonist	MDD and TRD	No dissociative or psychotomimetic effects
Dextromethorphan/ quinidine (Neudexta^®^)	Low-affinity NMDAR Antagonist and σ1	Approved for use in PBA. Possible development for MDD	Dizziness
Eliprodil, (EVT-101, ENS-101) ^4^	NR2B-selective NMDA antagonist	MDD and TRD	Generally well tolerated
D-cycloserine	NMDAR glycine site partial agonist	TRD	Without dissociation or psychotomimetic effects
Psilocybin formulations ^3^	5-HT_2A_ agonist	MDD and TRD	Psychedelic effects
GLYX-13 (Rapastinel^®^)	NMDA receptor modulator (i.v.)	TRD	Without dissociation or psychotomimetic effects
Gate-251 (AGN-241751) (Zelquistenel^®^)	NMDA receptor modulator (oral)	MDD and TRD	Without dissociation or psychotomimetic effects
Gate-202 (NRX-1074) (Apimostinel^®^)	NMDA receptor modulator (i.v.)	MDD and TRD	Without dissociation or psychotomimetic effects
Synthetic psychedelics ^3^	5-HT_2A_ agonist and other	TRD	Data needed to assess
TS-161	mGlu_2/3_ receptor prodrug oral	TRD	Without dissociation or psychotomimetic effects
TAK-653 (NBI-1065845) ^5^	AMPAR potentiator	MDD and TRD	Without dissociation or psychotomimetic effects

5-HT: 5-hydroxytryptamine or serotonin; AMPA: α-amino-3-hydroxy-5-methyl-4-isoxazolepropionic acid; ASIB: acute suicidal ideation and behavior; GABA: gamma amino butyric acid; MDD: major depressive disorder; mGlu: metabotropic glutamate; NMDA: N-methyl-D-aspartate; PBA: pseudobulbar affect; PTSD: post-traumatic stress syndrome; TRD: treatment-resistant depression. ^1^ Approved by US FDA in 2023. ^2^ Recent clinical trial findings for both (*R*)-ketamine and (*S*)-methadone have not met their sponsor-decided a priori clinical endpoints (see individual discussion of these compounds in text above). ^3^ See [9] for more details. ^4^ Development was discontinued in 2021. ^5^ Phase 2 data were just disclosed showing positive efficacy with primary and secondary endpoints in MDD patients with good tolerability.

**Table 2:** Potential therapeutic applications of (*R*)-ketamine as suggested from the experimental literature.

Disease	Rationale
MDD, BD, TRD	See Section 2
SUD	See Section 4
Pain	See inflammation below
Inflammation, colitis, sepsis	Rodent inflammation models—this Section
Delirium	Model using LPS-induced neural inflammation in mice—this Section
Autoimmune encephalomyelitis, MS	Model of experimental encephalomyelitis in mice (see [56])—this Section
PD	Link to depression and blockade of dopamine neurotoxicity (see [56])
AD and dementias	Link to depression (see [56])
Cognitive impairment	Link to depression using models of depression signs/cognitive impairment in mice— differentiation from (*S*)-ketamine—this Section
Schizophrenia	Model of maternal immune activation-induced neurobiological and behavioral alterations in mice—this Section
Developmental disorders	Model of maternal immune activation-induced neurobiological and behavioral alterations in mice—this Section
Stroke	Link to depression and effects in models and markers of stroke (see [56])
Anesthesia	Based upon use of racemic ketamine
Seizures	Based upon anti-seizure efficacy of racemic ketamine

AD: Alzheimer’s disease; LPS: lipopolysaccharide; MDD: major depressive disorder; MS: multiple sclerosis; PD: Parkinson’s disease; TRD: treatment-resistant depression.

**Table 3:** Side effect, tolerability, and safety differentiation of (*R*)-ketamine from (*R*,*S*)- and S-ketamine.

Effect	Differentiation	Data ^1^
Subjective	Little dissociation or psychotomimetic effects with (*R*)-ketamine	Human, rodent models, EEG
Sedation	Less with (*R*)-ketamine	Human, rodent models, EEG
Memory and cognition	Improvements with (*R*)-ketamine	Rodent models
Tolerance and sensitization	Reduced liability	Rodent models
Abuse liability	Reduced liability	Rodent models, human subjective reports

^1^ Data are summarized in this Section of the paper.

## Abbreviations

5-HT	5-hydroxytryptamine or serotonin
ADAMPA	Alzheimer’s diseaseα-amino-3-hydroxy-5-methyl-4-isoxazolepropionic acid
ASIB	acute suicidal ideation and behavior
BDNF	brain-derived neurotrophic factor
EEG	electroencephalogram
FDA	Food and Drug Administration
GABA	gamma amino butyric acid
GABAAR	GABA_A_ receptor
ICSS	intracranial self-stimulation
LPS	lipopolysaccharide
M-5MPEP	2-(2-(3-methoxyphenyl)ethynyl)-5-methylpyridine
mGlu	metabotropic glutamate
mPFC	medial prefrontal cortex
mTOR	mammalian target of rapamycin
MS	multiple sclerosis
NMDA	N-methyl-D-aspartate
PBA	pseudobulbar affect
PCP	phencyclidine
PFC	prefrontal cortex
REM	rapid eye movement sleep
nREM	non-rapid eye movement sleep
PD	Parkinson’s disease
PTSD	post-traumatic stress syndrome
SOC	standard of care
SUD	substance abuse disorder
TRD	treatment-resistant depression
